# p63 Expression Defines a Lethal Subset of Muscle-Invasive Bladder Cancers

**DOI:** 10.1371/journal.pone.0030206

**Published:** 2012-01-10

**Authors:** Woonyoung Choi, Jay B. Shah, Mai Tran, Robert Svatek, Lauren Marquis, I-Ling Lee, Dasom Yu, Liana Adam, Sijin Wen, Yu Shen, Colin Dinney, David J. McConkey, Arlene Siefker-Radtke

**Affiliations:** 1 Department of Urology, The University of Texas M. D. Anderson Cancer Center, Houston, Texas, United States of America; 2 The Graduate School of Biomedical Sciences (GSBS), University of Texas-Houston Health Sciences Center, Houston, Texas, United States of America; 3 Department of Urology, The University of Texas Health Science Center, San Antonio, Texas, United States of America; 4 Department of Biostatistics, The University of Texas M. D. Anderson Cancer Center, Houston, Texas, United States of America; 5 Department of Cancer Biology, The University of Texas M. D. Anderson Cancer Center, Houston, Texas, United States of America; 6 Department of Genitourinary Medical Oncology, The University of Texas M. D. Anderson Cancer Center, Houston, Texas, United States of America; Penn State Hershey Cancer Institute, United States of America

## Abstract

**Background:**

p63 is a member of the p53 family that has been implicated in maintenance of epithelial stem cell compartments. Previous studies demonstrated that p63 is downregulated in muscle-invasive bladder cancers, but the relationship between p63 expression and survival is not clear.

**Methodology/Principal Findings:**

We used real-time PCR to characterize p63 expression and several genes implicated in epithelial-to-mesenchymal transition (EMT) in human bladder cancer cell lines (n = 15) and primary tumors (n = 101). We correlated tumor marker expression with stage, disease-specific (DSS), and overall survival (OS). Expression of E-cadherin and p63 correlated directly with one another and inversely with expression of the mesenchymal markers Zeb-1, Zeb-2, and vimentin. Non-muscle-invasive (Ta and T1) bladder cancers uniformly expressed high levels of E-cadherin and p63 and low levels of the mesenchymal markers. Interestingly, a subset of muscle-invasive (T2–T4) tumors maintained high levels of E-cadherin and p63 expression. As expected, there was a strongly significant correlation between EMT marker expression and muscle invasion (p<0.0001). However, OS was shorter in patients with muscle-invasive tumors that retained p63 (p = 0.007).

**Conclusions/Significance:**

Our data confirm that molecular markers of EMT are elevated in muscle-invasive bladder cancers, but interestingly, retention of the “epithelial” marker p63 in muscle-invasive tumors is associated with a worse outcome.

## Introduction

Bladder cancers develop along two “tracks” producing tumors with very different clinical characteristics [1]. One leads to the formation of papillary tumors with high recurrence rates that rarely metastasize or cause death, whereas the other leads to the development of non-papillary, muscle-invasive tumors, of which a subset progresses rapidly and is fatal. At present it is impossible to prospectively identify the lethal muscle-invasive tumors; however, accumulating evidence suggests that molecular reprogramming characteristic of a developmental process known as epithelial-to-mesenchymal transition (EMT) is involved. Muscle-invasive cancers are characterized by downregulation of E-cadherin and p63, two “epithelial” markers uniformly expressed in normal urothelium and in non muscle-invasive cancers [2]–[5]. These changes are accompanied by upregulation of mesenchymal markers Zeb-1, Zeb-2, vimentin, and MMP9, leading to increased invasion and migration. A role for EMT in driving bladder cancer progression and metastases is consistent with a large body of evidence emerging in other solid tumors, particularly breast cancer [6]–[8]. Furthermore, enforced expression of EMT regulators can cause changes reminiscent of those observed in cancer stem cells [8]–[10]. Because cancer stem cells appear generally to be resistant to conventional chemotherapy, the link between EMT and “stemness” provides additional support for the idea that EMT contributes to lethality.

We initiated the present study to directly test the hypothesis that EMT markers could identify the subset of muscle-invasive tumors displaying aggressive clinical behavior. In human bladder cancer cell lines, we observed a direct correlation between E-cadherin and p63 expression, and an inverse correlation between these markers and Zeb-1, Zeb-2, and vimentin, indicating they cluster into unique “epithelial” and “mesenchymal” subsets. Our data confirm that mesenchymal markers (Zeb-1, Zeb-2, MMPs, and vimentin) are expressed almost exclusively by muscle-invasive tumors [11], [12], the subset of bladder cancer with worse outcome. Interestingly, our data also demonstrate that within this muscle-invasive subset, tumors retaining p63 (which appears to be an “epithelial” marker), display significantly worse survival than those muscle-invasive tumors displaying a more typical “mesenchymal” phenotype. We discuss possible explanations for the link between p63 and poor survival outcome.

## Results

### Expression of EMT markers in published gene expression datasets

**Figure 1:**
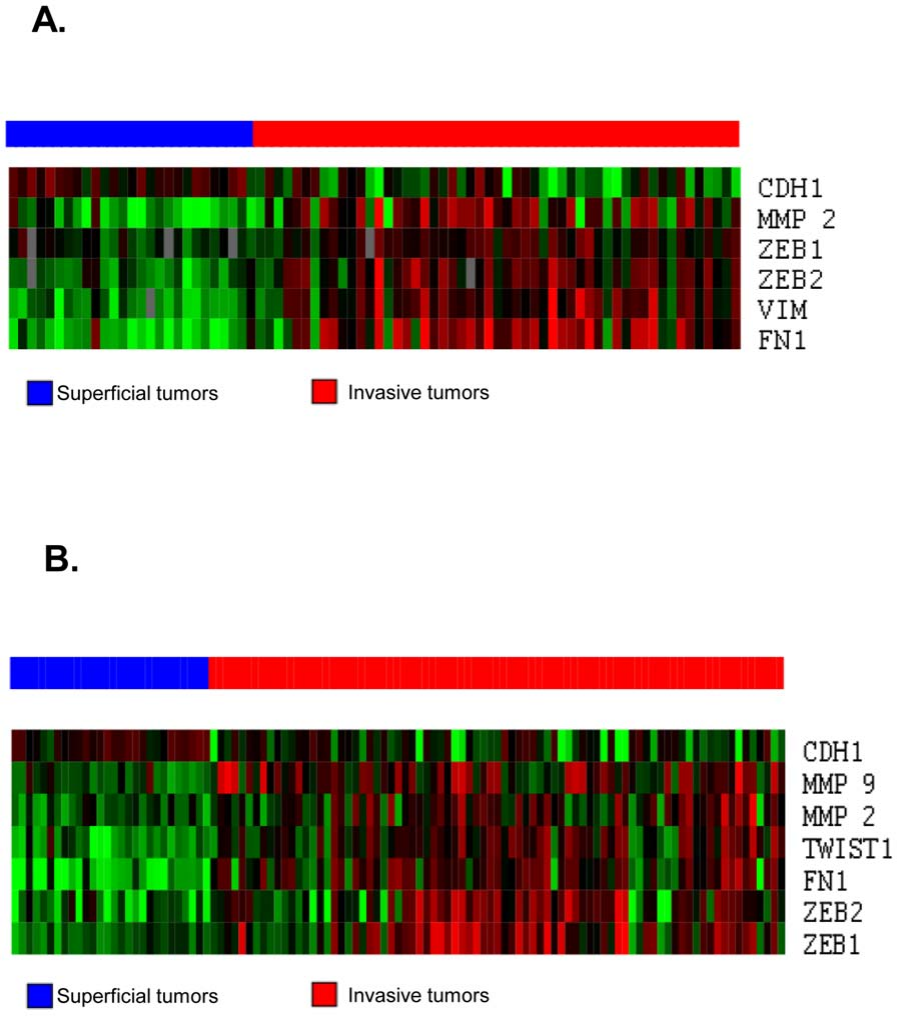
EMT marker expression in bladder cancers including primary tissue. A & B: All EMT marker genes with significant differences in expression (P<0.001) in 2 independent publicly available datasets. An in-house cDNA microarray with 10368 probes was used in A [13]. The HU-133A Affymetrix platform was used in B [14].

We first examined the expression of EMT markers in two publicly available gene expression profiling datasets [13], [14]. In both, unsupervised hierarchical clustering distinguished superficial from muscle-invasive disease [13], [14]. Superficial tumors expressed higher levels of “epithelial” marker E-cadherin than muscle-invasive tumors; conversely, muscle-invasive tumors expressed higher levels of “mesenchymal” markers (Figure 1A-B).

### Relationships among EMT markers in human bladder cancer cell lines

**Figure 2:**
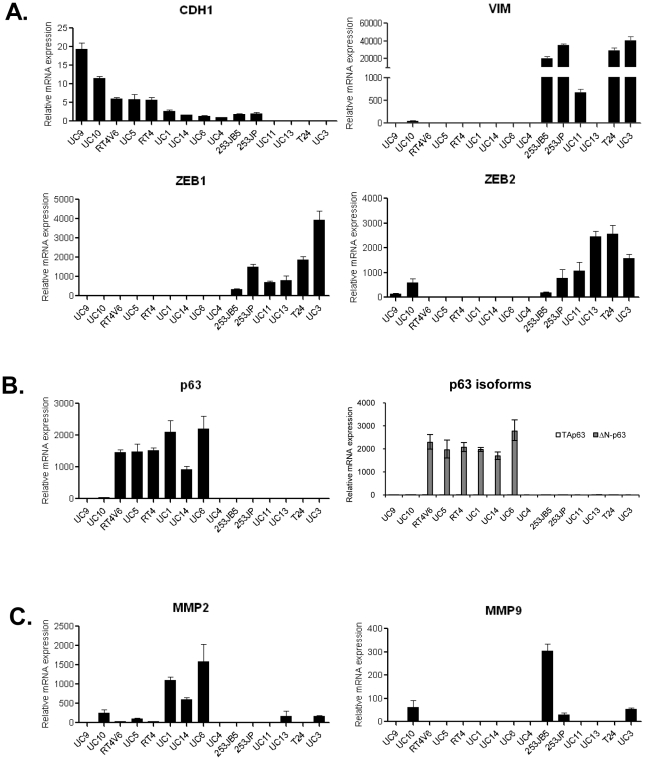
Differential expression of EMT markers in bladder cancer cell lines. (A) mRNA expression of E-cadherin, Zeb-1, Zeb-2 and vimentin were measured by quantitative real-time PCR. An inverse correlation between E-cadherin and Zeb-1/Zeb2 was observed across the cell lines. (B) mRNA expression of total p63, TAp63 and ΔN-p63 isoforms were measured by isoform specific primers. (C) Relative mRNA expression of MMP2 and MMP9 in bladder cancer cells. Data shown are mean ± SEM from triplicate samples.

We next measured the expression of EMT markers in 15 bladder cancer cell lines by quantitative real-time PCR. Results suggested the existence of 2 dichotomous subsets of tumors, one displaying an “epithelial” pattern characterized by expression of E-cadherin and p63; the other characterized by expression of “mesenchymal” markers Zeb-1, Zeb-2, and vimentin (Figure 2A–B). Quantification of both p63 mRNA (Figure 2B) and protein (data not shown) levels demonstrated it was expressed exclusively by the E-cadherin-positive, “epithelial” cell lines, and that expression of the ΔN-p63 isoforms exceeded expression of the TA-p63 isoforms by several orders of magnitude (Figure 2B). Contrary to our expectations, MMP2 and MMP9 levels were not closely associated with EMT in our cell lines (Figure 2C); however, the small number of cell lines for which the MMPs were elevated impacts the significance of this observation. Importantly, previous studies concluded that “mesenchymal” bladder cancer cells are as a group highly invasive and resistant to radiation and EGFR-directed therapy [11], [12], [15]–[17], suggesting they might possess unfavorable biological characteristics.

### EMT marker expression in a new set bladder tumors

**Figure 3:**
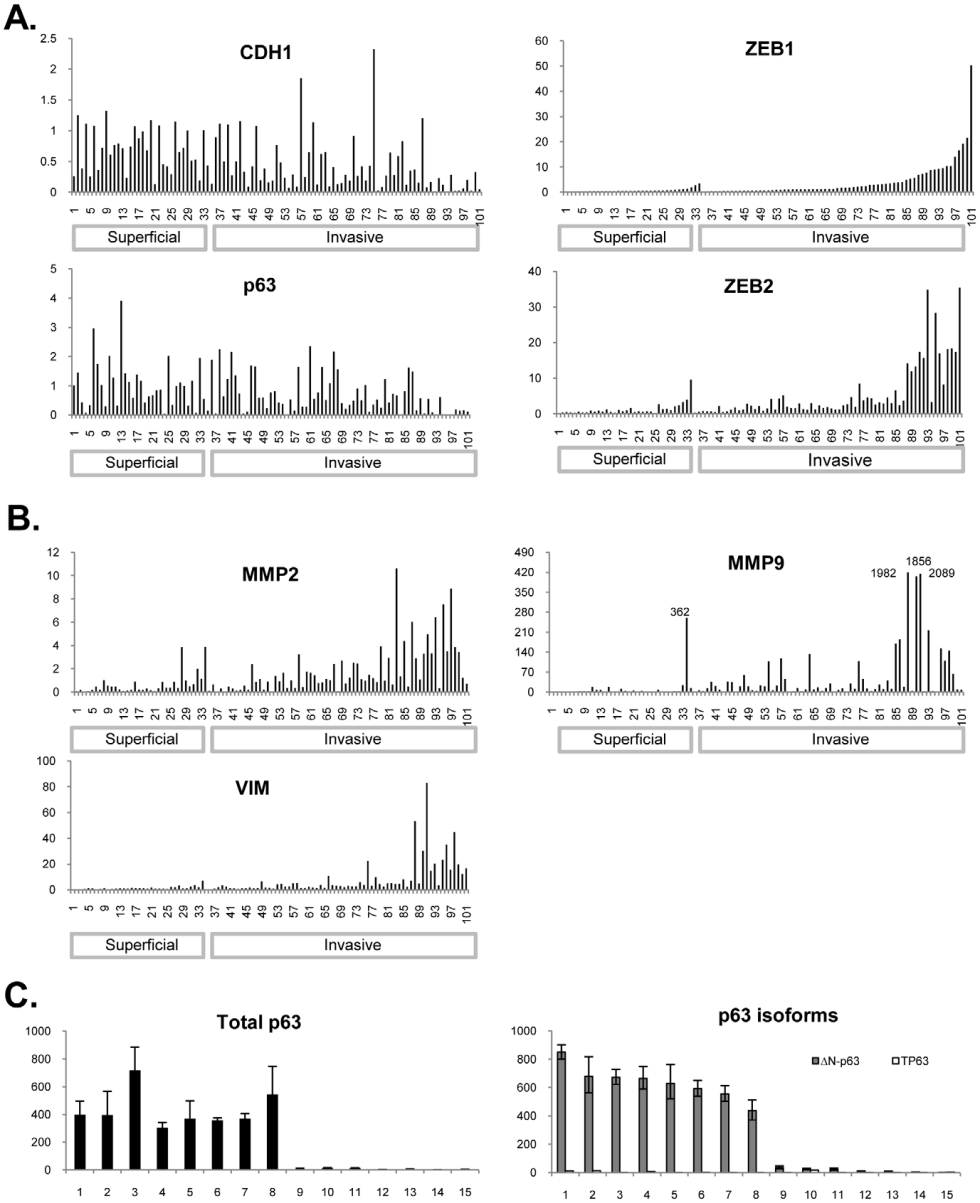
Expression of EMT markers in primary bladder tumors. (A) Expression of core E-cadherin-related EMT markers (E-cadherin, p63, Zeb-1, and Zeb-2) in 101 primary bladder tumors was assessed by quantitative real-time PCR. (B) Expression of MMP2, MMP9, and vimentin. (C) Expression of TAp63 and ΔN-p63 in a representative subset of the samples. All samples in each figure are shown in the same order.

We then measured the gene expression of EMT markers by RT-PCR in primary bladder cancers (n = 101, Table 1). Consistent with the public data (Figure 1) and other reports [11], [12], real time PCR data revealed that superficial tumors expressed higher levels of epithelial markers E-cadherin and p63, whereas muscle-invasive tumors expressed higher levels of mesenchymal markers (vimentin, Zeb1, Zeb2, MMP2, MMP9)(Figure 3 A and B). As shown in Figure 3, superficial tumors as a group more consistently expressed E-cadherin and p63 [3], [4], [18]–[23] than did muscle-invasive tumors (p-value<0.0001, Figure S1A). Similarly to our cell line data, ΔN-p63 was the major isoform of p63 present in primary tumor tissue (Figure 3C). Conversely, muscle-invasive tumors more frequently expressed the mesenchymal markers (Zeb-1, Zeb-2, vimentin) than did superficial tumors (all three p-values<0.0001, Figure S1B). As we had observed in cell lines, expression of E-cadherin was directly associated with expression of p63 (Spearman's correlation coefficient rho = 0.32, p = 0.0028) and inversely with expression of Zeb-1 (rho = −0.37, p<0.0001), Zeb-2 (rho = −0.48, p<0.0001), and vimentin (rho = −0.49, p<0.0001). On the other hand, although MMP2 and MMP9 were also expressed at higher levels in the muscle-invasive tumors compared to superficial tumors (both p-values<0.0001, Figure S1C), we did not observe as strong correlations between expression of MMP2 and MMP9 and expression of p63 in either the overall set of tumors (Spearman's correlation rho = −0.17, p = 0.08, between p63 and MMP9, and rho = −0.24, p = 0.01 between p63 and MMP2), or in the sub-group of muscle-invasive patients (rho = −0.15, p = 0.21, between p63 and MMP9, and rho = −0.23, p = 0.065 between p63 and MMP2). However, a strong inverse correlation between E-cadherin and MMP9, and MMP2 was still maintained (rho = −0.46, p<0.001 with MMP9, rho = −0.38, p<0.001 with MMP2) in the overall group, and the same associations were observed in muscle-invasive patients (rho = −0.45, p<0.001 with MMP9; rho = −0.39, p = 0.002 with MMP2).

**Table 1:** Clinicopathologic characteristics of bladder tumor samples for RT-PCR, N = 101.

	Number	%
Age at tissue collection		
Median	64	
Range	40–92	
Gender		
Female	27	(27%)
Male	74	(73%)
Race		
Caucasian	82	(81%)
African American	11	(11%)
Hispanic	6	(6%)
Asian	1	(1%)
Arabic	1	(1%)
Histology		
TCC	74	(73%)
Mixed*	27	(27%)
TCC with squamous	17	(17%)
TCC with micropapillary	3	(3%)
TCC with sarcomatoid	3	(3%)
TCC with small cell	2	(2%)
TCC with adenocarcinoma	1	(1%)
TCC with lymphoepithelioma	1	(1%)
Stage		
TaN0M0	14	(14%)
T1N0M0	20	(20%)
T2N0M0	6	(6%)
T3-4aN0M0	23	(22%)
T4b or N+ or M+	38	(38%)

*All cases of mixed histology were present in the invasive group except for one case of micropapillary in the non-invasive group for the RT-PCR.

### Higher p63 and MMP9 are associated with adverse OS in patients with muscle-invasive disease

**Figure 4:**
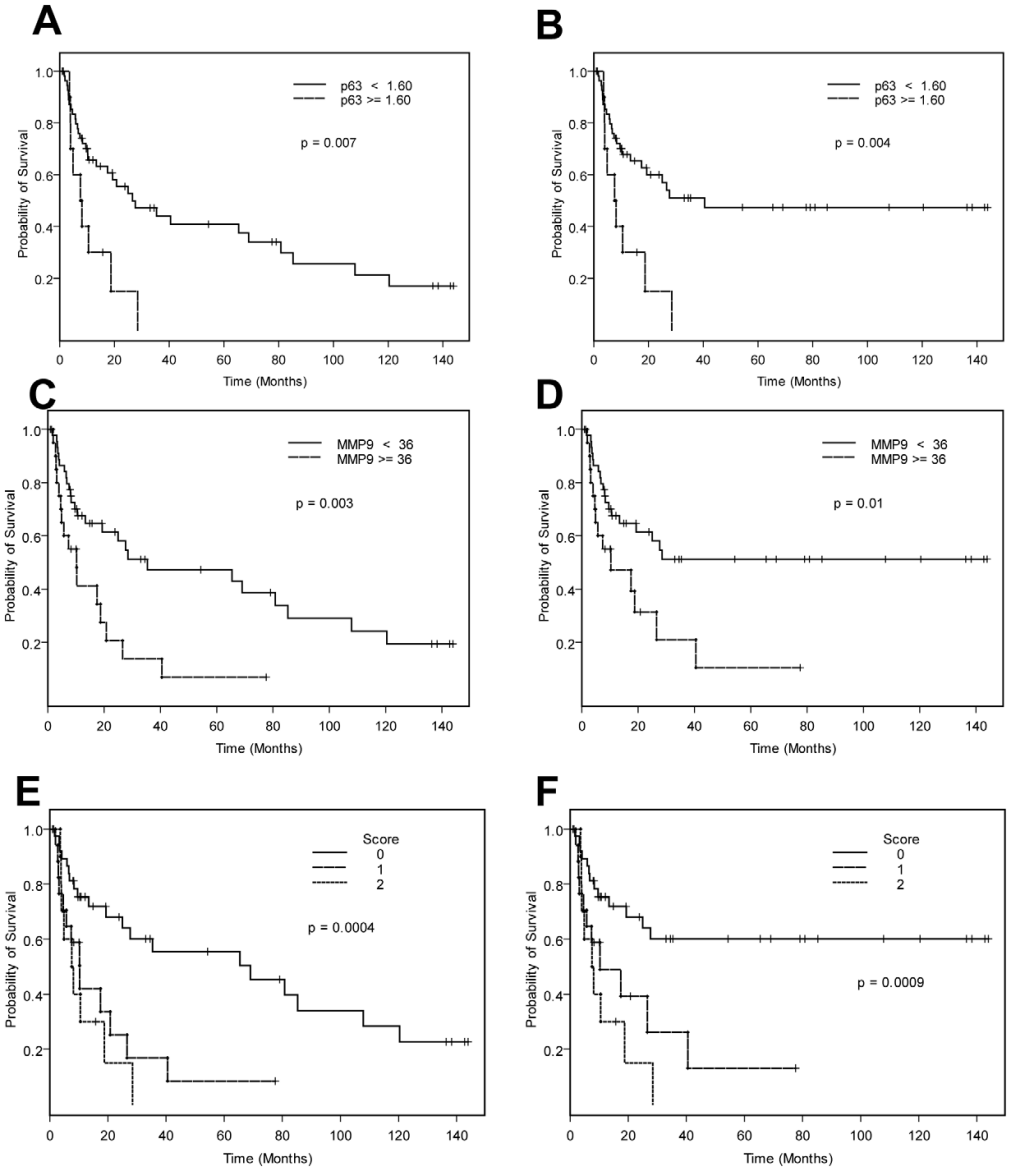
Kaplan-meier overall survival (OS) and disease-specific survival (DSS) curves based upon RT-PCR data for p63 and MMP9 in the subset of invasive tumors. All p-values provided are 2-sided, using the log-rank test. A: Elevated p63 was associated with adverse outcome in patients with muscle-invasive cancer (median OS 8 months, 95% confidence interval 4, 140 months), as compare to lower expression of p63 (median OS 27 months, 95% CI of 17, 85 months) log-rank p = 0.007. B: Elevated p63 was associated with adverse DSS in patients with muscle-invasive cancer (median DSS 8 months, 95% CI 19, 140), whereas patients with lower levels of p63 had an improved median DSS of 41 months (95% CI 19, 140+ months, log-rank p = 0.004). C: Elevated MMP9 was associated with adverse outcome (median OS 10.2 months, 95% CI i4.9, 26.6) as compared to lower MMP9 (median OS 35.4 months, 95% CI 19.3, 107.9, p = 0.003). D: Elevated MMP9 was associated with adverse DSS (median 10.3 months, 95% CI: 4.9, 140+) as compared to lower MMP9 (median DSS>140, 95% CI: 19.3, 140+ months, p = 0.01). E (OS) and F (DSS): Using a multivariate Cox regression model, p63 and MMP9 were independent predictors for adverse outcome in muscle-invasive disease. Patients were assigned 1 point each for either elevated p63 or MMP9 (0 = neither elevated, 1 = either elevated, 2 = both elevated). Worst survival was observed when both markers were elevated, and best when neither was elevated (median OS for 0, 1, or 2: > = 69, 10 months, and 8 months, with a 5-year OS or 55%, 8%, and 0% respectively).

Finally, we correlated EMT marker expression as measured by RT-PCR with survival. Since, as a group, patients with superficial tumors survive longer than those with muscle-invasive tumors, expression of an EMT phenotype characterized by loss of E-cadherin and p63 was associated with a poor prognosis, largely as a result of the ability to stratify patients between superficial and muscle-invasive disease (data not shown). However, when confined to the muscle-invasive subset of tumors, we found that retention of p63 expression was associated with adverse outcome (Figure 4A–B). The cutoff point for elevated p63>1.60 was obtained using regression tree analysis. Specifically, elevated p63 was associated with a significantly worse median OS of 8 months (95% confidence interval of 4, 140 months), whereas patients with lower levels of p63 had an improved median OS of 27 months (95% CI of 17, 85 months, log-rank p = 0.007). Analyses of the results for DSS outcomes were similar to those for OS outcome. Elevated p63 was associated with a median DSS of 8 months with a 95% confidence interval of (19, 140) months, whereas patients with lower levels of p63 had an improved median DSS of 41 months (95% CI: 19, 140+months, log-rank p-value = 0.004). Even in this muscle-invasive group, maintenance of p63 correlated directly with the expression of epithelial markers (E-cadherin rho = 0.34, p = 0.006), and inversely with mesenchymal markers (vimentin: rho = −0.35, p = 0.0047; zeb-1: rho = −0.36, p = 0.003; and zeb-2: rho = −0.39, p = 0.0014; respectively). Interestingly, retention of p63 in patients classified as having T1 tumors was also associated with significantly adverse survival outcome. Patients with T1 disease with higher levels of p63 had a median survival of 58 months, whereas there were no disease-related deaths in patients with lower levels of p63 in this subset (data not shown).

In a previous study we showed that a low E-cadherin/MMP9 ratio was also associated with an adverse survival outcome. Therefore, we also assessed the relationship between MMP9 expression and OS in this cohort (Figure 4C and D). Consistent with the previous data, high level expression of MMP9 was associated with shorter OS (median OS 10.2 months, 95% CI 4.9, 26.6) as compared to those tumors expressing lower amounts of MMP9 (median OS 35.4 months, 95% CI 19.3, 107.9, p = 0.003, Figure 4C). Likewise the median DSS was shorter with high-level expression of MMP9 (median DSS 10.3 months, 95% CI 4.9, 140+ months) and compared to those expressing lower amounts of MMP9 (median DSS>140, 95% CI 19.3, 140+ months, p = 0.01, Figure 4D).

We also confirmed the association between elevated p63 and poor survival in muscle-invasive urothelial cancer using an independent gene-expression dataset (GEO Accession number GSE13507 [24])Using regression-tree analysis, the cut-off point for p63 elevation was obtained. Elevated p63 was associated with a significantly worse OS and DSS (log-rank p = 0.012 and 0.002, respectively, Figure S2). Additional attempts at tissue confirmation using immunohistochemistry in a muscle-invasive bladder cancer tissue microarray were inconclusive, largely as a result of lack of specificity of the commercially available 4A4 antibody for ΔN-p63, a finding also confirmed by an outside group [25].

### p63 and MMP9 are independent predictors of OS

A multivariate Cox regression model was used to assess simultaneously the effect of expression of two or more EMT markers by RT-PCR and disease stage on OS. It is not surprising that disease stage was a statistically significant predictor of OS (HR = 2.15 invasive vs. superficial, p = 0.05). Interestingly, both continuous p63 and MMP9 were independent predictors of OS (p63: HR = 1.69 for p63, p = 0.004, log MMP9: HR = 1.25, p = .013) in addition to disease stage among all 101 patients. Other marker expressions were not associated with OS outcome in the multivariate model. Despite including other tumor characteristics, including presence/absence of squamous histology, disease stage, and age at diagnosis in the multivariate regression model, both p63 and the log of MMP9 remained as independent predictors of OS (p63: HR = 1.54, p = 0.017; log-MMP9: HR = 1.26, p = 0.011). Both p63 and the log transformation of MMP9 were also independent predictors of DSS (p63: HR = 2.04 for p63, p = 0.0006, log MMP9: HR = 1.31, p = .006) in addition to disease stage among all 101 patients. Including presence/absence of squamous histology, disease stage, and age at diagnosis in the multivariate regression model, both p63 and the log of MMP9 remained as independent predictors of DSS (p63: HR = 1.63, p = 0.022; log-MMP9: HR = 1.35, p = 0.003). When we restricted our multivariate marker analyses to the muscle-invasive tumors (n = 67, from the 101 samples used in RT-PCR), the same trend but slightly increased hazard ratios for OS with both p63 and MMP9 was observed (p63: HR = 2.17, p = 0.0003; log-MMP9: HR = 1.31, p = 0.007), suggesting that both p63 and MMP9 are independent predictors for adverse survival in muscle-invasive disease. The same trend was also observed with slightly increased hazard ratios for DSS (p63: HR = 2.40, p = 0.0018; log-MMP9: HR = 1.37, p = 0.003) as compared to OS. Assigning patients 1 point each for either elevated expression of p63 or MMP9 (0 = neither elevated, 1 = either elevated, 2 = both elevated), stratified survival with the worst survival when both were elevated (Figure 4E and F), and the best survival when neither was elevated (median OS for 0, 1, or 2: > = 69, 10 months, and 8 months, with a 5-year OS of 55%, 8% and 0% respectively). Proportional hazards assumptions for each risk factor is satisfied in the regression model for either 67 muscle-invasive patients, or 101 patients with primary bladder cancer.

## Discussion

Previous studies concluded that loss of p63 is associated with shorter survival in patients with bladder cancer [3], [20]. In contrast, our results suggest that maintenance of p63 expression is associated with adverse outcomes in patients with muscle-invasive bladder cancer. On the surface our results appear to contradict these other studies. However, we suspect this apparent discrepancy is due in part to the inclusion of superficial cancers displaying uniformly good survival characteristics in the earlier analyses as opposed to our current focus on their impact in muscle-invasive disease. As a group, the most superficial bladder cancers have excellent long-term survival (near 100%), and uniformly express high levels of E-cadherin and p63. On the other hand, loss of p63 is restricted to a subset of the muscle-invasive tumors, and muscle-invasive disease is typically associated with worse clinical outcomes as compared to superficial, non-invasive cancer. Therefore, inclusion of superficial tumors uniformly displaying an “epithelial” phenotype could dilute out an adverse effect of an “epithelial” phenotype in the muscle-invasive cohort. In addition, the commercial antibody (4A4) that is most commonly used to measure p63 in tissue sections cannot distinguish the two major p63 isoforms (TA and ΔN), so the relationship between ΔN p63 expression and poor outcome in muscle-invasive bladder cancers could not be recognized in previous IHC-based studies that employed this reagent [4], [20]. We attempted to confirm our mRNA-based results using the 4A4 on tissue microarrays and the results were inconclusive (data not shown). Consistent with this conclusion, while our manuscript was being prepared for submission Cordon-Cardo's group generated an anti-p63 antibody that is specific for the ΔN isoforms and used it on an independent cohort of muscle-invasive tumors to show that ΔN p63 protein levels also identify a lethal subset of cancers, whereas their results with the 4A4 antibody were inconclusive [25]. Ultimately prospective studies will be required to rigorously test the clinical significance of these findings.

Work performed over the past 5 years has renewed interest in the role of EMT in cancer progression and metastasis. One influential study demonstrated that isogenic metastatic variants of a murine breast cancer cell line overexpressed the E-cadherin repressor, Twist, and that elevated Twist expression was required for metastasis [7]. Subsequent work demonstrated that enforced Twist expression in normal mammary epithelial cells triggered EMT and simultaneously upregulated expression of mammary cancer stem cell markers [26]. Parallel studies by others have reinforced the notion that EMT drives tumor progression, metastasis, drug resistance, and a “stem-like” phenotype [10], [27]. With respect to bladder cancers, recent studies implicated EMT in disease progression, invasion/migration, and resistance to radiation and targeted therapy [11], [12], [15]–[17]. With these observations in mind, we expected that measuring EMT marker expression would identify the most lethal subset of muscle-invasive bladder cancers. Our results confirm that EMT markers were elevated in muscle-invasive cancers. Strikingly, however, increased p63 expression was associated with adverse outcomes both in muscle-invasive and in T1 tumors.

Several recent studies have shown that EMT and “stem-like” phenotype is regulated by micro RNAs (miR-205 and miR-200 family) [28]–[31]. miR-205 and the miR-200 family can maintain the epithelial phenotype by suppressing the expression of mesenchymal transcription factors (Zeb-1 and Sip1) and the introduction of miR-205 and miR-200 into mesenchymal cells can reverse mesenchymal cell morphology to an epithelial phenotype [30]. Interestingly, we have recently discovered that ΔNp63 directly promotes miR205 expression (M. Tran, manuscript in preparation), providing an explanation for the tight correlation between expression of ΔNp63 and epithelial markers in bladder cancer cell lines and primary tumors.

We are also investigating ΔNp63's effects on bladder cancer cell biology in order to better understand why its expression correlates with poor patient survival. Although structurally similar to p53, p63 is phylogenetically older than its cousin and displays much higher inter-species conservation [32], [33]. Furthermore, even though p53 and p63 interact with similar DNA sequence motifs, most p63 isoforms do not transactivate p53 target genes but rather can function as dominant negatives suppressing p53-dependent transactivation [33]. P63 is expressed at high levels in the basal layers containing the normal stem cell compartments in many different epithelial tissues, including the urothelium. Targeted ablation of p63 expression disrupts normal bladder differentiation in the mouse, leading to loss of the basal/suprabasal layer with selective retention of so-called “umbrella” cells [34]. These observations have led many investigators to propose that p63 is essential for the maintenance of self-renewal and/or survival of normal stem cells [35]. Thus, p63 expression in muscle-invasive disease may be associated with both epithelial phenotype and “stemness”[36]-[38]. However, cells within the normal urothelium express TAp63 (not ΔNp63), which would be expected to have very different effects on cell biology. Strikingly, our preliminary studies indicate that stable knockdown of ΔNp63 in bladder cancer cells causes strong inhibition of proliferation, effects that are associated with downreguliation of c-Myc mRNA and protein expression (L. Marquis et al, manuscript under revision), Therefore, we currently favor the idea that ΔNp63's significance as a negative prognostic marker is related to its effects on tumor cell proliferation. Additional mechanistic studies are required to define the relationships between the EMT phenotype, “stemness”, proliferation, drug sensitivity, and metastasis in preclinical models and primary tumors.

### Conclusions

Superficial (non muscle-invasive) tumors express higher levels of the epithelial markers E-cadherin and p63, while muscle-invasive tumors have higher expression of mesenchymal markers Zeb1, Zeb2, Vimentin, MMP2 and MMP9. Nonetheless, there is a subset of muscle-invasive cancer that has an adverse prognosis in the setting of elevated p63 expression. This elevation in p63 is associated with retained expression of E-cadherin and decreased levels of mesenchymal markers including vimentin, Zeb1 and Zeb2, suggesting that coupling between p63 expression and the “epithelial” phenotype remains present in a subset of muscle-invasive cancers. MMP9 expression appears to be independent from p63 in predicting adverse outcomes, suggesting regulation by independent mechanisms. The molecular mechanisms driving muscle invasion in tumors displaying an “epithelial” phenotype and the role of EMT in chemotherapy responsiveness and metastasis need to be more fully explored in future studies.

## Materials and Methods

Using public gene expression datasets we determined whether decreased expression of the “epithelial” marker E-cadherin and increased expression of several “mesenchymal” markers (the E-cadherin repressors Zeb-1, Zeb-2, and Twist, the matrix metalloproteases MMP2 and MMP9, fibronectin, and vimentin) was associated with progression to muscle-invasion. We then used a panel of bladder cancer cell lines to determine whether expression of “epithelial” and “mesenchymal” markers were mutually exclusive displaying distinct “epithelial” and “mesenchymal” phenotypes. Using quantitative real-time PCR we tested whether the relationship between EMT and muscle-invasive disease was present in a new cohort of patients with bladder cancer and correlated EMT marker expression with survival.

### Analysis of EMT Markers in Public Gene Expression Profiling Datasets

Two public human bladder cancer gene expression datasets were used to analyze EMT markers. Data from Blavari et al and Carbayo et al were downloaded from Gene Expression Omnius (GEO Accession number GSE-1827 [13]) and the Journal of Clinical Oncology [14], respectively. BRB ArrayTools version 4.2, developed by NCI [39] was used to analyze the data [13], [14]. To select genes differentially expressed between the two different sub-groups (superficial and muscle-invasive tumors), a class comparison tool within BRB ArrayTools was used to calculate the significance of the observations (ie: *P*<0.001 computed from two-sample t-test with a false discovery rate (FDR) less than 0.1). To observe patterns of EMT gene expression, each gene's value, adjusted to a mean of zero, was used for clustering with Cluster and TreeView [40].

### Cell lines

Cell lines were obtained from the MD Anderson Bladder SPORE Tissue Bank, and their identities validated by DNA fingerprinting using AmpFlSTR® Identifiler® Amplification (Applied Biosystems, Foster City, CA) or AmpF*l*STR® Profiler® PCR Amplification (Applied Biosystems), performed by the MD Anderson Characterized Cell Line Core, or ourselves, respectively. These fingerprints are available on the MD Anderson Bladder SPORE website. Cell lines were cultured in modified Eagle's MEM supplemented with 10% fetal bovine serum, vitamins, sodium pyruvate, L-glutamine, penicillin, streptomycin, and nonessential amino acids at 37°C in 5% CO_2_ incubator.

### Human specimens

Clinical outcomes data and patient samples for real-time PCR were obtained for 101 patients from the MD Anderson genitourinary cancers research database. Fresh frozen tissues were obtained from our SPORE Tissue Core. Samples were collected using macrodissection focusing on areas with at least 80% tumor concentration. All patients had previously signed informed consent allowing collection of their tissue and clinical data in our genitourinary research database. An additional IRB approved protocol was obtained for the specific analyses described in this paper. All tissue samples were reviewed by a pathologist. Patients were classified as muscle-invasive for tumor growth into the muscularis propria; otherwise, they were classified as superficial (non muscle-invasive). Total RNA from human specimens were isolated using mirVana™miRNA isolation kit (Ambion, Inc).

### Real-time Reverse Transcriptase PCR analysis

EMT markers were analyzed by Taqman-based real-time PCR (ABI PRISM 7500; Applied Biosystems). The comparative CT method [41] was used to determine relative gene expression for each target gene; the cyclophilin A gene was the internal control used to normalize the amount of amplifiable RNA (Assay ID numbers: E-cadherin; Hs00170423_m1, ZEB1; Hs00232783_m1, ZEB2; Hs00207691_m1, Vimentin; Hs00185584_m1, MMP2; Hs00234422_m1, MMP9; Hs00957562_m1 and Cyclophilin A; Hs99999904_m1). Primers and fluorescent probes for p63 isoforms were designed by Primer Express 3.0 (Applied Biosystems) with the following sequences: TAP63 forward primer 5′-TGCAGGACTCGGACCTGAGT-3′, reverse primer 5′- TGTTCAGGAGCCCCAGGTT-3′, probes 5′-ACCCCATGTGGCCAC-3′; ΔN-p63 forward primer 5′-GGAAAACAATGCCCAGACTCA-3′, reverse primer 5′- TGTTCAGGAGCCCCAGGTT-3′, probes 5′-TTTAGTGAGCCACAGTACAC-3′. Primers and probes for p63 isoforms were validated using standard curve methods (data not shown).

### Statistical Methods

Statistical analyses were based on information from 101 patients. The primary objectives were to examine correlations between marker expression and disease stage, and to evaluate the association between marker expression and overall survival (OS) and disease-specific survival (DSS). Disease stage was stratified as superficial (Ta or T1) or muscle-invasive (> = T2). Expression values were compared between superficial and muscle-invasive bladder cancer using the Wilcoxon rank sum test. Correlations among expression of markers were quantified using Spearman's rho coefficients. The Kaplan-Meier estimate of survival distribution was displayed by the investigated biomarker expression characterized as high and low (e.g. p63, MMP9), where the cutoff point to define high and low was obtained from regression tree analyses [42]. The log-rank test was used to compare survival distributions between groups. The proportional hazards model was used to assess the effects of multiple markers and tumor stage on DSS and OS. We performed a logarithmic transformation of the MMP9 expression values due to the skewedness of the data. All p-values presented are 2-sided.

## Supporting Information

Figure S1 EMT marker expression in primary bladder tumors was measured by quantitative real-time PCR and stratified by patient stage as superficial (Ta or T1), or muscle-invasive (> = T2). A: The epithelial markers E-cadherin (CDH1) and p63 were elevated in superficial tumors. B: The mesenchymal markers Zeb1, Zeb2, and vimentin were expressed at higher levels in invasive as compared to superficial tumors. C: MMP2 and MMP9 were also expressed at higher levels in invasive tumors.
(TIF)

Figure S2 Elevated p63 was associated with worse prognosis in an independent gene-expression dataset. A: Overall Survival. Median OS not reached in low p63, and 13 months in high p63 (log-rank p = 0.012). B. Disease-specific survival. Median DSS not reached in low p63, and 15.1 months in high p63 (log-rank p = 0.002).
(DOCX)
