# Supplementary figures and images for: p63 Expression Defines a Lethal Subset of Muscle-Invasive Bladder Cancers
Source: PLoS One. 2012 Jan 10;7(1):e30206. doi: 10.1371/journal.pone.0030206 (PMC3254658; doi:10.1371/journal.pone.0030206)

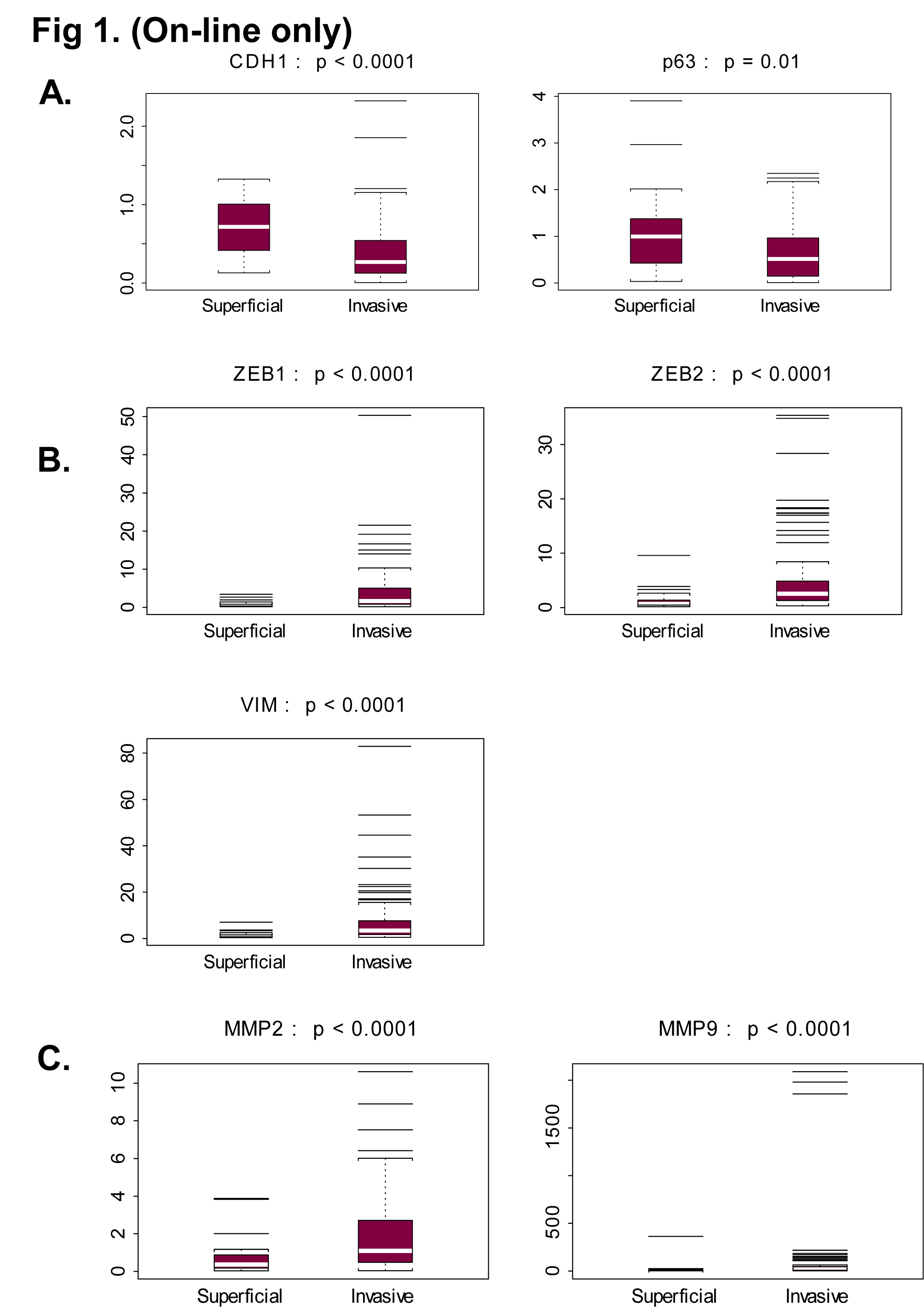

Supplement: Figure S1 — EMT marker expression in primary bladder tumors was measured by quantitative real-time PCR and stratified by patient stage as superficial (Ta or T1), or muscle-invasive (> = T2). A: The epithelial markers E-cadherin (CDH1) and p63 were elevated in superficial tumors. B: The mesenchymal markers Zeb1, Zeb2, and vimentin were expressed at higher levels in invasive as compared to superficial tumors. C: MMP2 and MMP9 were also expressed at higher levels in invasive tumors. (TIF) [file pone.0030206.s001.tif]
