# Supplementary material for: p63 Expression Defines a Lethal Subset of Muscle-Invasive Bladder Cancers
Source: PLoS One. 2012 Jan 10;7(1):e30206. doi: 10.1371/journal.pone.0030206 (PMC3254658; doi:10.1371/journal.pone.0030206)
Supplement: Figure S2 — Elevated p63 was associated with worse prognosis in an independent gene-expression dataset. A: Overall Survival. Median OS not reached in low p63, and 13 months in high p63 (log-rank p = 0.012). B. Disease-specific survival. Median DSS not reached in low p63, and 15.1 months in high p63 (log-rank p = 0.002). (DOCX) [file pone.0030206.s002.docx]

Supplemental Figure 2a.

Supplemental Figure 2b.
